# Supplementary material for: Epidemiology and Trends over Time of Foreign Body Injuries in the Pediatric Emergency Department
Source: Children (Basel). 2021 Oct 19;8(10):938. doi: 10.3390/children8100938 (PMC8534431; doi:10.3390/children8100938)
Supplement: Supplementary file 1 [file children-08-00938-s001.zip › children-1403968-supplementary.pdf]

Supplementary Material

Table S1. Descriptive characteristics of children with FB presentations to the pediatric emergency department of Padova. Continuous data are reported as median (I, III quartiles), categorical data are re-ported as absolute numbers (percentages). The chi square test or Fisher exact test, whatever appropriate, has been performed.

			**Infant toddler (< 2 y)**	**Pre-schooler (< 6 y)**	**Schooler (> 6 y)**	**Overall**	**P-Value**
			(N=1112)	(N=980)	(N=992)	(N=3084)	Statistic
**Gender**	Female	3084	50% (555)	43% (422)	40% (398)	45% (1375)	<0.001
	Male	-	50% (557)	57% (558)	60% (594)	55% (1709)	
**Foreign Body type**	No-food	591	70% (172)	73% (137)	54% (85)	67% (394)	<0.001
	Food	-	30% (75)	27% (51)	46% (71)	33% (197)	
**Foreign Body site**	Ears, nose throat	2566	46% (427)	53% (444)	37% (298)	46% (1169)	<0.001
	Gastrointestinal	-	50% (468)	39% (328)	30% (238)	40% (1034)	
	Eyes	-	4% (33)	8% (71)	33% (259)	14% (363)	
**Outcome**	Death	3084	0% (0)	0% (0)	0% (0)	0% (0)	<0.001
	Discharged home	-	91% (1007)	95% (927)	95% (943)	93% (2877)	
	Admitted	-	8% (94)	4% (38)	4% (38)	6% (170)	
	Other	-	1% (11)	2% (15)	1% (11)	1% (37)	

Table S2

Incidence Rate (IR) table according to the year and clinical characteristics (sex, nationality, foreign body type, triage color and FB location).

The absolute number of FB injuries events have been reported with 95% Poisson Confidence interval for IR over 1000 resident population. Percentages (%) of FB injury events over PED presentations have also been reported with a 95% confidence interval. Univariate GLM Poisson P-values for the relation between time and FB incidence had been indicated.

Year	2007	2008	2009	2010	2011	2012	2013	2014	2015	2016	2017	2018	p-value
**Female**													
FB Events	106	99	131	114	121	108	105	108	111	96	135	141	
IR	9 (7.44,10.79)	8.36 (6.87,10.08)	10.83 (9.13,12.76)	9.32 (7.76,11.11)	9.81 (8.22,11.64)	8.77 (7.27,10.5)	8.45 (6.99,10.15)	8.63 (7.15,10.33)	8.8 (7.31,10.51)	7.68 (6.29,9.28)	10.99 (9.29,12.92)	11.53 (9.78,13.5)	0.283
% ED Accesses	1.4 (1.17,1.7)	1.3 (1.05,1.56)	1.7 (1.44,2.01)	1.5 (1.22,1.75)	1.6 (1.31,1.87)	1.4 (1.2,1.74)	1.5 (1.2,1.76)	1.4 (1.2,1.75)	1.6 (1.31,1.9)	1.3 (1.08,1.6)	1.6 (1.34,1.87)	1.6 (1.37,1.89)	0.422
**Male**													
FB Events	148	111	119	164	162	152	122	140	127	143	141	180	
IR	11.82 (10.07,13.8)	8.81 (7.32,10.53)	9.29 (7.77,11.03)	12.66 (10.87,14.67)	12.52 (10.73,14.51)	11.73 (10.01,13.67)	9.31 (7.8,11.03)	10.36 (8.79,12.15)	9.38 (7.89,11.08)	10.66 (9.06,12.48)	10.6 (8.99,12.42)	13.72 (11.86,15.8)	0.341

% ED Accesses	1.5 (1.3,1.79)	1.1 (0.93,1.35)	1.2 (1.01,1.44)	1.8 (1.47,1.99)	1.6 (1.41,1.91)	1.6 (1.39,1.9)	1.3 (1.11,1.57)	1.4 (1.22,1.69)	1.3 (1.14,1.6)	1.5 (1.26,1.75)	1.2 (1.06,1.47)	1.5 (1.33,1.78)	0.796
**Italian**													
FB Events	225	172	215	220	231	206	179	193	183	181	208	254	
IR	10.61 (9.31,12.04)	8.17 (7.04,9.43)	10.33 (9.04,11.76)	10.71 (9.39,12.17)	11.38 (10,12.89)	10.01 (8.74,11.43)	8.85 (7.64,10.19)	9.68 (8.41,11.09)	9.26 (8.02,10.65)	9.29 (8.03,10.69)	10.72 (9.36,12.23)	13.21 (11.69,14.89)	0.08
% ED Accesses	1.6 (1.38,1.79)	1.2 (1.03,1.39)	1.6 (1.37,1.78)	1.6 (1.42,1.85)	1.7 (1.51,1.95)	1.6 (1.38,1.81)	1.4 (1.22,1.64)	1.47 (1.28,1.69)	1.5 (1.28,1.7)	1.4 (1.21,1.62)	1.4 (1.18,1.55)	1.6 (1.42,1.81)	0.747
**Not Italian**													
FB Events	29	38	35	58	52	54	48	55	55	58	68	67	
IR	9.36 (6.53,13.06)	11.21 (8.18,15.05)	8.53 (6.14,11.57)	12.48 (9.66,15.89)	10.46 (7.99,13.49)	11.5 (8.82,14.76)	9.07 (6.85,11.81)	9.05 (6.96,11.59)	8.6 (6.61,11.02)	9.03 (6.99,11.5)	10.98 (8.67,13.74)	10.93 (8.62,13.7)	0.8
% ED Accesses	1.0 (0.7,1.4)	1.2 (0.8,1.6)	0.9 (0.7,1.3)	1.5 (1.2,2)	1.3 (1,1.6)	1.4 (1.1,1.9)	1.3 (1,1.7)	1.3 (1,1.7)	1.4 (1,1.8)	1.4 (1.1,1.9)	1.5 (1.2,1.9)	1.5 (1.2,1.9)	0.011
**Food**													
FB Events	21	12	17	16	14	17	12	19	21	12	16	20	

IR	0.86 (0.57,1.27)	0.49 (0.28,0.81)	0.68 (0.43,1.04)	0.64 (0.39,0.98)	0.55 (0.33,0.88)	0.67 (0.42,1.03)	0.47 (0.27,0.77)	0.73 (0.47,1.09)	0.8 (0.53,1.18)	0.46 (0.27,0.76)	0.63 (0.39,0.97)	0.79 (0.51,1.17)	0.987
% ED Accesses	8.3 (5.47,12.31)	5.7 (3.3,9.72)	6.8 (4.29,10.62)	5.8 (3.57,9.14)	4.9 (2.97,8.13)	6.5 (4.12,10.22)	5.3 (3.05,9.01)	7.7 (4.96,11.65)	8.8 (5.84,13.11)	5.0 (2.9,8.57)	5.8 (3.6,9.21)	6.2 (4.07,9.43)	0.733
**No food**													
FB Events	25	27	36	43	35	34	30	37	34	34	25	34	
IR	1.03 (0.7,1.47)	1.1 (0.76,1.56)	1.45 (1.05,1.95)	1.71 (1.27,2.25)	1.38 (1,1.88)	1.35 (0.96,1.83)	1.18 (0.83,1.63)	1.42 (1.03,1.92)	1.3 (0.93,1.77)	1.31 (0.94,1.79)	0.98 (0.66,1.4)	1.34 (0.96,1.83)	0.866
% ED Accesses	9.8 (6.8,14.1)	12.9 (9,18.1)	14.4 (10.6,19.3)	15.5 (11.7,20.2)	12.4 (9,16.7)	13.1 (9.5,17.7)	13.2 (9.4,18.2)	14.9 (11,19.9)	14.3 (10.4,19.3)	14.2 (10.4,19.2)	9.1 (6.2,13)	10.6 (7.7,14.4)	0.503
**Colour code: Yellow**													
FB Events	64	46	58	66	43	39	47	55	42	53	42	58	
IR	2.63 (2.06,3.32)	1.88 (1.41,2.46)	2.33 (1.8,2.96)	2.62 (2.06,3.29)	1.7 (1.27,2.25)	1.54 (1.13,2.06)	1.84 (1.39,2.4)	2.11 (1.63,2.71)	1.61 (1.19,2.13)	2.04 (1.57,2.63)	1.64 (1.22,2.17)	2.29 (1.77,2.91)	0.093
% ED Accesses	2.4 (1.9,3.07)	1.5 (1.12,1.98)	1.8 (1.4,2.33)	2.2 (1.75,2.81)	1.6 (1.22,2.2)	1.5 (1.1,2.05)	1.6 (1.2,2.11)	1.7 (1.34,2.26)	1.5 (1.11,2.02)	1.6 (1.25,2.13)	1.9 (0.95,1.73)	1.7 (1.31,2.17)	0.017

**Colour code: Red**													
FB Events	4	0	0	3	2	8	2	1	3	0	1	3	
IR	0.16 (0.07,0.36)	0.0 (0.0, 0.0)	0.0 (0.0, 0.0)	0.12 (0.04,0.29)	0.08 (0.02,0.22)	0.32 (0.16,0.57)	0.08 (0.02,0.22)	0.04 (0.01,0.14)	0.11 (0.04,0.28)	0.0 (0.0, 0.0)	0.04 (0.01,0.14)	0.12 (0.04,0.28)	0.691
% ED Accesses	2.3 (0.9,5.8)	0.0 (0.0,2.3)	0.0 (0.0,3.3)	2.3 (0.8,6.7)	1.5 (0.4,5.4)	5 (2.6,9.6)	1.8 (0.5,6.2)	0.8 (0.1,4.3)	2.2 (0.8,6.4)	0.0 (0,2.4)	0.6 (0.1,3.5)	1.8 (0.6,5.1)	0.703
**Colour code: Green**													
FB Events	162	133	166	176	198	190	152	168	155	148	181	217	
IR	6.67 (5.72,7.73)	5.44 (4.59,6.4)	6.66 (5.73,7.71)	6.99 (6.03,8.06)	7.83 (6.82,8.96)	7.52 (6.52,8.62)	5.95 (5.08,6.94)	6.46 (5.55,7.47)	5.93 (5.07,6.9)	5.71 (4.86,6.67)	7.07 (6.12,8.14)	8.56 (7.5,9.74)	0.162
% ED Accesses	1.4 (1.2,1.7)	1.2 (1,1.4)	1.4 (1.2,1.7)	1.5 (1.3,1.8)	1.7 (1.5,2)	1.8 (1.5,2)	1.4 (1.2,1.6)	1.4 (1.2,1.6)	1.3 (1.1,1.6)	1.3 (1.1,1.5)	1.3 (1.2,1.5)	1.5 (1.3,1.7)	0.809
**Colour code: White**													
FB Events	24	31	26	33	40	23	26	24	38	38	52	43	
IR	0.99 (0.67,1.42)	1.27 (0.9,1.75)	1.04 (0.71,1.48)	1.31 (0.93,1.79)	1.58 (1.16,2.11)	0.91 (0.61,1.32)	1.02 (0.7,1.45)	0.92 (0.62,1.33)	1.45 (1.06,1.95)	1.47 (1.07,1.97)	2.03 (1.55,2.62)	1.7 (1.26,2.24)	0.003

% ED Accesses	0.8 (0.5,1.1)	1.1 (0.7,1.5)	1 (0.7,1.5)	1.2 (0.9,1.7)	1.2 (0.9,1.6)	0.7 (0.5,1.1)	1.2 (0.8,1.7)	1.3 (0.9,1.9)	2.0 (1.4,2.7)	1.8 (1.3,2.4)	1.9 (1.4,2.4)	1.7 (1.3,2.3)	
**Foreign body location: Ear, nose, throat**													
FB Events	83	72	80	87	113	94	85	96	92	96	127	144	
IR	3.41 (2.76,4.19)	2.95 (2.34,3.66)	3.21 (2.58,3.95)	3.45 (2.8,4.22)	4.47 (3.72,5.33)	3.72 (3.04,4.51)	3.33 (2.69,4.07)	3.69 (3.02,4.46)	3.52 (2.87,4.27)	3.7 (3.04,4.48)	4.96 (4.17,5.86)	5.68 (4.83,6.64)	
% ED Accesses	32.7 (27.2,38.67)	34.3 (28.2,40.94)	32.0 (26.53,38.02)	31.3 (26.13,36.97)	39.4 (34.39,45.73)	36.2 (30.55,42.16)	37.4 (31.41,43.9)	38.7 (32.86,44.9)	38.7 (32.7,44.98)	40.2 (34.16,46.49)	46.0 (40.23,51.91)	44.8 (39.51,50.33)	
**Foreign body location: Gastrointestinal**													
FB Events	83	65	87	104	88	90	80	88	84	75	79	111	
IR	3.41 (2.76,4.19)	2.66 (2.09,3.34)	3.49 (2.83,4.26)	4.13 (3.41,4.96)	3.48 (2.83,4.25)	3.56 (2.9,4.33)	3.13 (2.52,3.86)	3.38 (2.75,4.12)	3.21 (2.6,3.93)	2.89 (2.31,3.58)	3.09 (2.48,3.8)	4.38 (3.64,5.23)	0.597
% ED Accesses	32.7 (27.2,38.7)	31.0 (25.1,37.5)	34.8 (29.2,40.9)	37.4 (31.9,43.2)	31.1 (26,36.7)	34.6 (29.1,40.6)	35.2 (29.3,41.7)	35.5 (29.8,41.6)	35.3 (29.5,41.6)	31.4 (25.8,37.5)	28.6 (23.6,34.2)	34.6 (29.6,39.9)	0.765
**Foreign body location: Eyes**													
FB Events	41	40	43	37	27	31	24	25	21	23	28	23	

IR	1.69 (1.25,2.24)	1.64 (1.2,2.18)	1.73 (1.28,2.28)	1.47 (1.07,1.98)	1.07 (0.74,1.51)	1.23 (0.87,1.69)	0.94 (0.63,1.35)	0.96 (0.65,1.37)	0.8 (0.53,1.18)	0.89 (0.59,1.29)	1.09 (0.76,1.54)	0.91 (0.61,1.31)
% ED Accesses	16.1 (12.1,21.2)	19.0 (14.3,24.9)	17.2 (13,22.4)	13.3 (9.8,17.8)	9.5 (6.6,13.5)	11.9 (8.5,16.4)	10.6 (7.2,15.2)	10.1 (6.9,14.5)	8.8 (5.8,13.1)	9.6 (6.5,14)	10.1 (7.1,14.3)	7.2 (4.8,10.5)

Abbreviations: ED, emergency department, FB, foreign body, IR, incidence rate
